# Microfluidic Paper-Based Devices at the Edge of Real Samples: Fabrication Limits, Hybrid Detection, and Perspectives

**DOI:** 10.3390/mi17010105

**Published:** 2026-01-13

**Authors:** Hsing-Meng Wang, Sheng-Zhuo Lee, Lung-Ming Fu

**Affiliations:** Department of Engineering Science, National Cheng Kung University, Tainan 701, Taiwan; n98144028@gs.ncku.edu.tw (H.-M.W.); szlee0729@gmail.com (S.-Z.L.)

**Keywords:** microfluidic paper-based analytical devices, paper-based sensors, microfluidic paper sensors, paper-based electrochemical devices

## Abstract

Microfluidic paper-based analytical devices (µPADs) convert ordinary cellulose into an active analytical platform where capillary gradients shape transport, surface chemistry guides recognition, and embedded electrodes or optical probes translate biochemical events into readable signals. Progress in fabrication—from wax and stencil barriers to laser-defined grooves, inkjet-printed conductive lattices, and 3D-structured multilayers—has expanded reaction capacity while preserving portability. Detection strategies span colorimetric fields that respond within porous fibers, fluorescence and ratiometric architectures tuned for low abundance biomarkers, and electrochemical interfaces resilient to turbidity, salinity, and biological noise. Applications now include diagnosing human body fluids, checking food safety, monitoring the environment, and testing for pesticides and illegal drugs, often in places with limited resources. Researchers are now using learning algorithms to read minute gradients or currents imperceptible to the human eye, effectively enhancing and assisting the measurement process. This perspective article focuses on the newest advancements in the design, fabrication, material selection, testing methods, and applications of µPADs, and it explains how they work, where they can be used, and what their future might hold.

## 1. Introduction

Microfluidic paper-based analytical devices (μPADs) have attracted sustained interest as a platform for decentralized sensing. Their appeal stems from cellulose itself: a material that is porous, inexpensive, mechanically compliant, and intrinsically hydrophilic. Fluid motion is governed by capillary forces rather than pumps, allowing microliter-scale assays to proceed autonomously across patterned channels. In places where laboratory infrastructure is limited or continuous monitoring is essential, μPADs introduce a form of microfluidics that exchanges the sophistication of the hardware for the sophistication of the material design. From environmental surveillance to clinical diagnostics and food inspection, the field has expanded beyond proof-of-concept toward translational applications [1,2].

Adoption of μPADs has accelerated, but practical use continues to be limited by material behavior, analytical reliability, and scale-up challenges. Paper substrates, despite low cost and passive wicking, show intrinsic variability in fiber arrangement, pore structure, and moisture response that undermines reproducibility across batches and suppliers. Such variability complicates scalable manufacturing, undermines storage stability, and propagates into inconsistent flow behavior, reagent dispersion, and channel definition once devices move beyond benchtop fabrication [3]. Under realistic sampling conditions, analytical limits become more obvious. Complex matrices—including whole blood, viscous food extracts, and contaminated environmental waters—introduce background coloration, protein adsorption, particulate blockage, and matrix-specific signal attenuation. Such influences narrow usable signal windows and weaken quantitative accuracy, reinforcing the gap between laboratory validation and dependable performance under field conditions [4].

However, paper resists the regularity associated with glass, silicon, or synthetic polymers. Fibers curl and take up moisture in patches; pores close, reopen, and drift with humidity, so techniques that look precise on engineered substrates lose their footing here. Wax finds its path through those gaps: once melted, it threads along microchannels and then stiffens, forming borders that corral liquid in a manner that feels almost accidental but seldom fails [1,2]. Tools capable of tighter lines—laser cutting, inkjet droplets, and photolithographic masks—draw thinner channels, yet every gain arrives with a toll in cost, reagent tolerance, or repeatability [3,4]. Past that point, geometry is no longer the main lever. Nanocellulose tightens the weave; hydrophobic coatings calm unwanted wicking; hybrid sheets accept carbon inks or metal particles as if they had always been part of the paper [5,6,7]. Stability improves, and so do signals, while mass production becomes slower and storage less forgiving.

Detection determines whether a μPAD merely reacts or truly informs. Colorimetric zones dominated the early landscape because anyone, anywhere, could immediately read a single spot—darkened, brightened, or shifted toward blue or red. Real samples unsettle that ideal—blood tones, plant pigments, and cloudy matrices blur the palette. Optical paths broaden the toolkit: carbon dots, lanthanide complexes, and perovskite emitters respond within narrow fiber pockets, where tiny analyte volumes produce signals far larger than expected [8,9,10,11]. Brightness becomes the marker rather than hue. Electrochemical formats provide an answer from a different perspective. Printed or laser-formed carbon electrodes monitor electron flow, largely indifferent to color or turbidity [5]. Enzymes, aptamers, and nanozymes depress detection limits, enabling several targets to be interrogated on the same sheet. Hybrid layouts knit optical and electrochemical cues together, preserving fidelity when sample matrices refuse to behave [12,13,14].

Field applications demonstrate how μPADs operate beyond controlled laboratories. Patterned cellulose absorbs metals, nitrates, and volatile species from soil or water and answers with shifts in tone, brightness, or conductivity that require no bench instruments [15,16,17]. Clinical use demands steadier hands; paper quietly separates erythrocytes, draws plasma through narrow veins of fiber, and holds amplification or immunoassay chemistries long enough to flag viral, bacterial, or oncologic markers. Wearable forms—patches, dental guards, even mask inserts—track sweat electrolytes, saliva metabolites, or aerosol signatures during everyday motion [18,19,20]. Food testing brings its urgency: a droplet from milk or seafood can expose adulterants or spoilage before shipment leaves the dock [21,22,23]. Agricultural residues ask for finer discrimination; organophosphates and neonicotinoids often hide in extracts, and electrochemical inhibition layers or aptamer films sort them out while humidity and storage history pull the fibers in unpredictable directions [24,25,26,27].

Artificial intelligence (AI) has started to modify the analytical role of μPADs by shifting emphasis from visual recording to computational interpretation. Smartphone cameras now function as quantitative sensing elements, with trained models extracting chemically relevant spectral cues rather than simple color contrast. Spatial irregularities—such as pixel clustering, edge darkening, and illumination gradients—are reformulated as numerical features suitable for concentration inference [22,28,29]. More advanced learning architectures can handle pigment drift and uneven reagent distribution, which makes it possible to see signal changes that would be hard to see by hand. Increasingly, computational insight feeds back into device engineering: simulated wicking profiles, flow-guided electrode layouts, and kinetic models for reagent delivery reduce reliance on trial-and-error experimentation and accelerate design refinement [26]. Most AI-assisted readout schemes remain tied to carefully curated datasets, controlled lighting, and standardized device layouts, causing accuracy to deteriorate once fabrication variability, ambient illumination, or unfamiliar sample matrices depart from trained conditions. In addition, most implementations confine algorithmic intervention to downstream signal interpretation. Few implementations address material heterogeneity, flow instability, and reagent distribution upstream, which limits robustness and hinders transferability outside controlled or centralized measurement environments [25].

This article focuses on four main research directions for μPADs that have seen rapid development recently. The discussion begins with the fabrication of μPADs. Their evolution has moved from wax-patterned channels to laser ablation, inkjet-derived geometries, and nanoscale coatings that stabilize reagents while embedding conductive structures. Next, detection has progressed from simple color patches to optical probes, electrochemical interfaces, and hybrid modes capable of handling real matrices. Next, this article will introduce how these infrastructures support applications such as environmental monitoring, distributed diagnostics, food safety testing, and pesticide residue monitoring. Finally, AI is improving image interpretation, reducing variability in fabrication, and directing device architecture toward scalable and reliable deployment.

## 2. Fabrication Techniques for μPAD

Fabrication of μPADs often begins by choosing the paper, because the fibers decide how a droplet behaves. Along narrow pathways, the liquid creeps forward, pulled by capillary tension, and the cellulose rarely calls for special coatings. The next decisions involve where to block the flow and where to let it pass: a wax line that refuses to wick, a cut that follows the grain of the sheet, or a window reserved for electrodes or reagents. A practical design benefits from hydrophobic borders that resist seepage, from channels that do not curl at the edges, and from sensing regions that remain sharply defined. Real complications appear in places that are difficult to predict: a patch of dense fibers that slows a front, a reagent band that blurs during drying, or a lamination step that shifts alignment by a fraction of a millimeter. Reliable fabrication grows from recognizing these small departures from the plan and adjusting the process until the paper, reagents, and geometry settle into a repeatable form.

### 2.1. Paper Substrates and Material Behaviors

In paper microfluidics, liquid propagation arises from spontaneous imbibition within intertwined cellulose fibers, where interfacial energetics dominate transport behavior rather than externally imposed pressure. Under simplified and well-defined conditions, capillary wicking along these fibrous pathways conforms to the Lucas–Washburn description [30,31], predicting a penetration length that increases with the square root of time and scales with effective pore radius, surface tension, and wetting affinity, while diminishing with increasing viscosity. Although idealized, this formulation establishes a useful baseline by linking contact angle, permeability, and apparent flow rate in μPAD architectures.

Real paper substrates, however, rarely conform to such ideal assumptions. Fiber orientation, pore-size distributions, tortuosity, and local compression create strong spatial heterogeneity. Moisture-induced fiber swelling and progressive reagent accumulation further reshape permeability dynamically during operation, rather than remaining fixed material properties [32]. Consequently, channels constructed to identical specifications may demonstrate noticeable discrepancies in flow velocity, reagent dispersion, and reaction timing—an effect that is especially pronounced in multilayer architectures or elongated flow paths. Variability inherent to fibrous substrates has therefore repositioned material modification as a central design lever rather than a peripheral adjustment. Hydrophobic or amphiphilic modifications alter surface energetics in ways that reshape local wetting dynamics and damp instabilities at the advancing front [2]. Regulation of permeability arises not only from chemical adjustment but also from structural reorganization of the pore network—via nanocellulose blending, mechanical compaction, or selective infiltration—thereby enabling programmable delays in fluid transport [33]. Such discrepancies become most evident in viscous biofluids and particle-bearing environmental samples, conditions under which fluid transport no longer conforms to the simplifying assumptions underlying classical capillary scaling.

**Figure 1:**
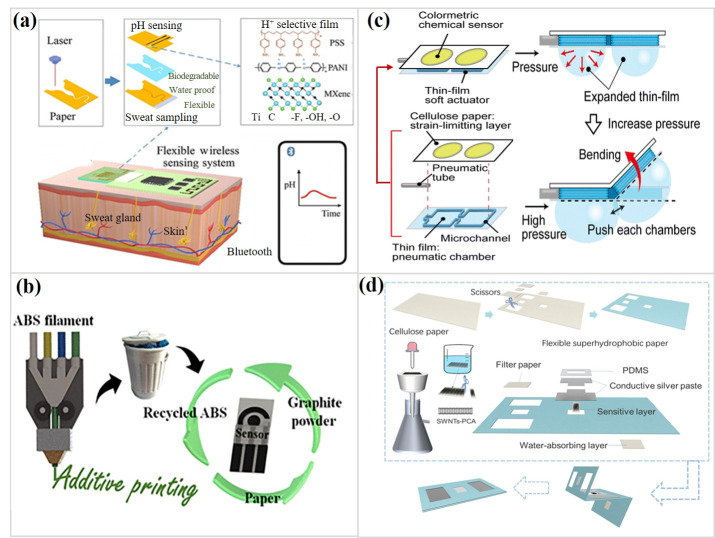
(**a**) Schematic illustration of the multilayer MXene all-paper-based sensor for continuous sweat pH detection. (Reprinted with permission from ref. [35]. Copyright 2025 ACS). (**b**) Schematic representation of the 3D-printed residues for fabrication of disposable paper-based electrochemical sensors. (Reprinted with permission from ref. [47]. Copyright 2023 ACS). (**c**) Schematic diagram of a microfluidic paper-based analytical soft actuator and expansion of the pneumatic chambers. (Reprinted with permission from ref. [51]. Copyright 2025 RSC). (**d**) Schematic illustration of the fabrication process of a flexible hydrophobic paper-based microfluidic field-effect sensor. (Reprinted with permission from ref. [44]. Copyright 2025 MDPI).

Subtle changes in surface chemistry often decide how such modified chips behave in practice. In metal–organic frameworks, lattice organization creates ordered cavities where adsorbed biomolecules preferentially localize near sites of catalytic or photonic significance, producing markedly sharpened local responses within confined pockets [34]. MXene-based paper also keeps its electrical conductivity over a wide range of biofluid pH levels (Figure 1a) [35]. However, gradual reagent aging and small differences between batches can still affect long-term signal stability. These examples show that the reliability of transport and the fidelity of signals in μPADs are not only determined by the nominal geometry but also by carefully controlled interfacial and chemical heterogeneity at the material level.

### 2.2. Patterning of Hydrophobic Barriers

Microchannel formation is often the first decisive act in μPAD fabrication. Shaping it into regions that invite fluid and those that remain dry transforms the sheet into a two-phase architecture. Once this contrast is established, capillary motion no longer drifts randomly along fibers; the path is steered by hydrophobic borders that protect reagent distribution and stabilize reaction timing.

Fabrication strategies approach the problem from different angles. Wax printing [29] relies on molten wax seeping into fiber junctions. After cooling, the wax solidifies into barriers that tolerate rapid prototyping and instructional assays [36,37]. Edge thickening can occur during melting, especially in layered assemblies, but its simplicity continues to make it attractive. Inkjet and chemical deposition [38] take a more deliberate route. Graphite, gold nanoparticles, or hydrophobic polymers are deposited at defined depths within the matrix, yielding sharply bounded channels. This localized placement supports cortisol detection with minimal reagent waste on fully printed platforms [39]. Automated PGMEA dispensing produces heat-free barrier networks suitable for ion analysis or blood typing [40], whereas spray or dip coatings [41] treat entire sheets uniformly. Their success depends on evaporation dynamics and the extent to which reagents remain anchored during drying [42].

Laser ablation and mechanical cutting impose geometry directly. CO_2_-etched motifs that undergo secondary hydrophobization enable electrochemical sweat patches on paper [30]. Microgrooves shaped by blade pressure can accelerate or slow capillary flow, granting control across hour-scale windows [43,44,45]. Excessive energy risks fiber carbonization and reduces biochemical activity, an outcome often missed until testing. Vapor-phase hydrophobization does not require direct contact; cyanoacrylate vapors create barriers in minutes. The technique is a practical method for use outside of traditional labs, but the depth of penetration and thickness of the coating are affected by the humidity in the air [46].

Three-dimensional printing [47,48] expands this toolkit (see Figure 1b). UV-curable resin infiltrates pores and cures into crisp hydrophobic ridges, and minor adjustments in curing time determine whether leakage or collapse occurs [49]. Extruded carbon-black filaments further reshape the concept, creating thermoplastic electrodes that sustain continuous-injection amperometry under radial flow, sidestepping conventional channel-barrier logic [50]. Table 1 compares the advantages, disadvantages, and applicability of different μPAD manufacturing technologies.

### 2.3. Multilayer Structural

Within two-dimensional μPAD configurations, lateral transport is readily regulated; however, available surface area diminishes quickly as reaction regions, rinsing operations, and multiplexed features are layered onto the same plane. Adding a vertical dimension redistributes capillary pathways and reorganizes analytical operations at the device level. In multilayer μPADs, stacked paper sheets are linked by through-layer openings or overlaps, allowing fluid to move vertically while remaining confined from adjacent lateral paths. Stacked μPAD architectures demand high registration fidelity. Alignment is typically guided by fiducial references, machined vias, and lamination fixtures, while localized bonding pressure helps preserve pore integrity. Subtle lateral misalignment can deform vertical connections, producing blockage, irregular wetting, or interlayer interference, and revealing the mechanical sensitivity inherent to multilayer assemblies [42].

Multilayer μPADs are built around two distinct structural logics. Adhesive stacking puts dimensional fidelity and secure sealing first. This feature makes vertical interconnects stable and flow timing predictable, but it also adds extra interfaces that make it harder to recycle and limit vapor exchange. Origami-based formats do not use any adhesives at all. Instead, functional areas are made separately and then connected by folding, which creates enclosed reaction pockets or staged flow paths. This simplifies alignment and speeds prototyping, but folded junctions remain mechanically delicate, with small misfolds readily leading to leakage or interlayer bleeding during prolonged wetting [42].

Recent developments step away from rigid multilayer constructs by taking advantage of paper’s intrinsic mechanical flexibility. In pneumatic paper actuators (see Figure 1c), paper coupled with elastomeric chambers bends in a controlled manner as applied pressure generates strain imbalance across the layered structure. Under these conditions, paper operates simultaneously as a fluidic carrier and a mechanical boundary, enabling sensing regions to conform to curved or irregular biological and environmental surfaces. This capability directly addresses a long-standing limitation of conventional μPADs [51].

Hydrophobically embossed paper operates through a distinct actuation mechanism, in which localized embossing paired with selective hydrophobic patterning converts moisture uptake into anisotropic swelling across the sheet thickness. As water enters predefined regions, differential expansion generates reversible bending or curling without electrical stimulation. This way, capillary absorption is directly turned into mechanical motion, which lets paper devices move sensing elements or control contact areas on their own. Such behavior recasts paper as an active, energy-storing material rather than a passive wicking substrate [19]. Taken as a whole, stacking strategies and mechanically adaptive elements move paper microfluidics past flat channel layouts, combining vertical flow with controlled deformation to access fluid–structure behaviors that single-layer formats cannot reach.

### 2.4. Electrode and Sensor Integration

The transport behaviors described and the spatial reconfiguration strategies outlined in Section 2.2 and Section 2.3 ultimately converge at the sensing interface. In electrochemical μPADs, analytical performance is finalized not by fluid delivery alone, but by how electrodes, recognition elements, and paper substrates are integrated into a continuous functional pathway. Integration, in this context, refers to more than physical attachment; it encompasses electrical continuity, mechanical compatibility, and chemical communication across heterogeneous domains within the device. At the most established level, screen-printed carbon electrodes remain a practical cornerstone. Their ability to bend, change humidity, and be handled many times keeps the signal stable in point-of-care settings, which makes them a favorable fit for multilayer or foldable paper architectures [52,53]. Such robustness allows these electrodes to function reliably as terminal readout nodes for capillary-driven assays introduced earlier in this section.

More sophisticated integration approaches move past electrodes as passive conductors and instead encode molecular selectivity within their architecture. In conductive paper modified with molecularly imprinted polymers, chemical recognition is converted directly into electrical contrast, enabling target binding to be read out without auxiliary labels and distributing the sensing function across the electrode surface itself [41]. A related challenge arises when electrical continuity must be preserved across chemically dissimilar paper regions. Continuous single-walled carbon nanotube (SWNT) networks (see Figure 1d) have been shown to bridge hydrophobic barriers and hydrophilic sensing zones, sustaining charge transport across patterned substrates. When interfaced with Mg^2+^-responsive DNAzyme gates, localized cleavage events regulate nanotube conduction, producing spatially confined, field-directed electrical responses [44]. These examples show that integration is a dynamic link between molecular changes and electronic networks on a mesoscale.

Mechanical adaptability substantially broadens integration routes, particularly for μPADs designed to interface with the body or irregular environments. Conductive threads stitched or embroidered into paper function as compliant electrical conduits, preserving signal continuity during bending or motion and enabling continuous metabolite monitoring during tissue regeneration or electrolyte transport under dynamic conditions [52]. At the sensing interface, the analytical outcome is shaped primarily by nanoscale spatial organization rather than bulk composition. Local electromagnetic confinement can be markedly intensified by gold-modified mesoporous MXene networks, enabling surface-enhanced Raman scattering (SERS) signals approaching 10^6^ while remaining fully compatible with porous paper supports [35,54]. Simultaneously, under controlled thermal and electrochemical conditions, MnO nanosheets formed within cellulose matrices retain catalytic activity by suppressing delamination and transport bottlenecks [55]. These material approaches show that deliberate nanoscale organization can overcome substrate heterogeneity, repositioning paper platforms as mechanically compliant and functionally active analytical systems.

## 3. Detection Techniques for μPAD

Detection strategy selection determines how a μPAD translates physical and chemical events into readable information, with direct consequences for analytical performance, device design, user operation, and robustness under non-ideal, real-world conditions. Early paper-based assays relied almost exclusively on binary color changes. Contemporary systems, by contrast, increasingly support quantitation, multiplexing, and spatially resolved readouts. In practice, five detection families dominate current designs: colorimetric, electrochemical, fluorescence-based, distance-encoded or instrument-free formats, and Raman/SERS-based sensing. Each offers distinct strengths, yet none is free from constraints, and recent research has focused as much on overcoming these limitations as on expanding analytical scope.

**Figure 2:**
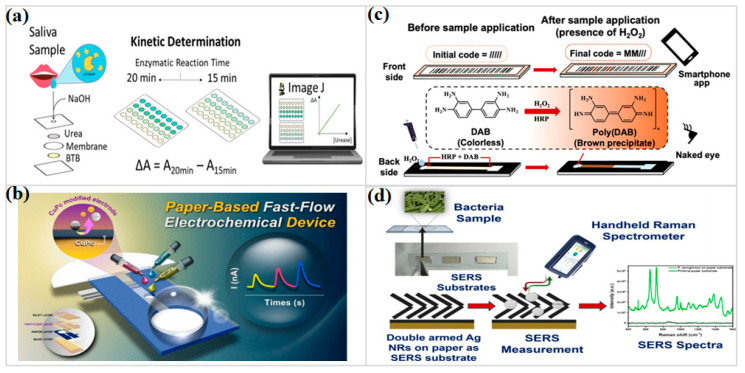
(**a**) Schematic representation of the colorimetric method of μPAD for monitoring urease activity in saliva. (Reprinted with permission from ref. [59]. Copyright 2025 MDPI). (**b**) Schematic illustration of the paper-based electrochemical device enabling high-throughput NADPH measurements. (Reprinted with permission from ref. [68]. Copyright 2025 ACS). (**c**) Schematic diagram of a distance-encoded PAD for enzymatic H_2_O_2_ and glucose detection. (Reprinted with permission from ref. [76]. Copyright 2025 ACS). (**d**) Schematic illustration of the paper-based SERS sensor for bacterial detection. (Reprinted with permission from ref. [77]. Copyright 2024 ACS).

Colorimetric detection remains the most accessible and widely deployed modality [56,57,58,59,60,61,62]. Its principal advantage lies in immediacy: a visible hue change can guide decisions in the field without instrumentation. However, this simplicity comes with well-known limitations. Ambient lighting, paper background heterogeneity, and subjective visual interpretation limit quantitative reliability, particularly in colored or protein-rich matrices. Recent work has therefore shifted from single-zone readouts toward structured designs that encode information spatially or temporally. RNAzyme-based assays targeting stress-associated miRNA408 in tomato samples demonstrate that enzymatic selectivity can be retained within visually minimalist μPAD formats [57]. In parallel clinical implementations, stacked and gas-diffusion designs compartmentalize volatile reaction products, allowing reproducible urease quantification in saliva for chronic kidney disease (CKD) screening, even under storage-induced variability (Figure 2a) [59]. Other approaches embed sample pretreatment directly into the paper network. Protein precipitation zones that are fixed upstream dispose of serum macromolecules before color development, turning the substrate into a passive filtration and reaction system [60]. Distance-encoded enzyme assays for branched-chain amino acids further reduce reliance on color intensity by translating reaction extent into spatial length, mitigating illumination and observer bias while retaining chemical specificity [62].

Electrochemical readout reshapes quantitative analysis by treating redox activity as an electrical event rather than an optical contrast [63,64,65,66,67,68]. On paper substrates, this translation preserves sensitivity even when samples receive minimal conditioning, although stability rarely comes for free. Signal drift commonly traces back to electrode fouling, gradual mechanical wear, or small inconsistencies introduced during printing, all of which accumulate over repeated use. Attention has therefore shifted toward how the sensing interface is built and maintained. Molecularly imprinted films formed on Pt single-atom catalysts supported by carbon polyhedra allow phenol discrimination in wastewater while remaining functional under dense ionic backgrounds [64]. MXene-based papers and melanin-derived coatings reinforce electron pathways and tolerate repeated deformation [65,66]. Folding-based immunosensors reduce the distance over which analytes must travel, but the analytical gain is tightly coupled to how precisely the layers are aligned during assembly [67]. Signal persistence is instead more reliably sustained in hybrid fast-flow designs, where periodic electrolyte exchange alleviates stagnation effects and supports repeated nicotinamide adenine dinucleotide phosphate (NADPH) readout without opening or reconfiguring the device (Figure 2b) [68].

Fluorescence readouts become indispensable once analytical sensitivity must surpass the limits of unaided vision [69,70,71,72]. Practical performance, however, is often shaped less by fluorophore brightness than by gradual photobleaching, intrinsic cellulose autofluorescence, and the stability of excitation conditions. Recent work shifts the burden of stability away from illumination control and toward how fluorescence is referenced and spatially confined. Quantum-dot-functionalized paper pads demonstrate how fluorescence contrast can remain readable during histamine analysis in canned tuna, even when illumination is uneven or poorly controlled [58]. In dual-emission upconversion systems, molecularly imprinted frameworks act upstream of optical readout, concentrating Sudan dyes in a manner that keeps fluorescence ratios steady even as excitation conditions drift [69]. Paper substrates coupled directly to living cultures capture lactate at the moment of release, thereby avoiding the dilution inherent to bulk collection schemes commonly used in cell-based assays [71]. In pathogen assays, spatial arrangement becomes decisive: bacteriophages anchored in a head-first orientation present tail fibers in a uniform geometry, so fluorescence amplification emerges consistently once Klebsiella pneumoniae is trapped within confined microreaction grids [72].

Instrument-free, distance-encoded readouts shift quantification away from signal intensity and toward spatial displacement along the substrate [73,74,75,76]. Although numerical resolution is reduced, these formats remain reliable under conditions where electronic support offers little practical value. DNA hydrogel valves illustrate this trade-off clearly: aflatoxin B1 alters capillary resistance in a concentration-dependent manner, converting travel length into a metric largely immune to illumination and user bias [75]. In multilayer strips patterned as barcodes, spatial information replaces intensity as the dominant readout cue. Discrete reaction zones can be captured reliably by smartphone cameras, reducing interpretive variability during hydrogen peroxide and glucose analysis (Figure 2c) [76]. The same spatial logic, however, loses resolving power at trace concentrations, where positional differences compress, a limitation that has steered recent designs toward coupling enzymatic amplification with distance-based encoding.

Raman and SERS-based sensing occupy the high-sensitivity end of the spectrum [77,78,79,80]. Their appeal lies in molecular fingerprinting and ultra-low detection limits, yet reproducible hotspot generation and substrate scalability remain persistent challenges. Advances increasingly focus on substrate architecture rather than solely on nanoparticle composition. Silver nanorods made by glancing-angle deposition turn cellulose into a uniform plasmonic scaffold that can tell the difference between pathogens even when there are only a few copies (Figure 2d) [77]. Bioinspired alveolar paper structures increase hotspot density for antifungal detection while preserving capillary transport [78]. Nanocellulose papers have shifted the practical detection floor for neurodegeneration-related biomarkers into the femtogram range, a gain that traces back to tightly regulated fibril spacing and the accompanying suppression of background scattering [79]. A parallel effect appears in biopolymer-treated substrates, where plasmonic nanoparticles are held in mechanically and chemically stable environments, supporting label-free glucose detection while extending usable shelf life [80]. Detection in μPADs is no longer defined by a single signal modality but by how limitations are actively engineered around. Current progress reflects a shift from asking whether paper can support a given detection technique to how paper geometry, surface chemistry, and flow architecture can be co-designed to offset each method’s inherent constraints.

Beyond established colorimetric and fluorescence schemes, a range of less orthodox detection routes illustrates how μPAD development has shifted from signal presentation toward problem-specific design [81,82,83,84,85,86,87]. These approaches rarely pursue novelty in readouts alone. Instead, each is framed around a limitation already exposed by classical assays. Luminescent probes, for instance, are adopted less for brightness enhancement than for their ability to buffer photon output against matrix coloration and scattering—persistent obstacles for intensity-based optics in heterogeneous samples [84]. Materials such as Ag-doped Cu_1−__x_Ag_x_S nanoparticles pack several chromogenic roles into one phase, cutting down reagent duplication and curbing the lateral spread that tends to blur multiplexed paper assays [85]. Under these conditions, paper no longer acts as a neutral support but actively confines reaction geometry. The same logic underpins paper-spray ionization mass spectrometry, which links the porous matrix directly to high-resolution analysis and sidesteps optical or electrochemical transduction altogether, enabling rapid metabolite profiling in three-dimensional tumor models where gradients are weak and transient [86]. Taken together, these routes point to a broader reorientation in μPAD design: detection strategies are increasingly engineered as targeted responses to defined analytical bottlenecks rather than interchangeable signal outputs, with paper operating simultaneously as reactor, separator, and transducer. Table 2 compares the advantages, disadvantages, and applicability of different μPAD detection methods.

**Table 2:** Overview of the comparison of μPAD detection methods.

Detection Method and Ref.	Advantages	Limitations	Representative Applications
Colorimetric [56,57,58,59,60,61,62]	● Extremely low cost and intuitive visual interpretation ● Requires minimal instrumentation ● Works with a wide range of chemical and enzymatic assays ● Good compatibility with capillary-driven fluidics	■ Limited sensitivity for trace analytes ■ Influenced by ambient light and user perception ■ Narrow dynamic range ■ Target cross-reactivity in complex matrices	▲ Rapid screening of ions, metabolites, and biomarkers ▲ Food safety testing ▲ Environmental monitoring of pollutants and nutrients
Fluorescence [69,70,71,72]	● Higher sensitivity than colorimetry ● Ratiometric or dual-channel designs reduce noise ● Compatible with molecular probes	■ Photobleaching and background autofluorescence ■ Requires excitation source and detector ■ Probe immobilization stability can vary	▲ Quantification of amino acids, antibiotics, hormones ▲ Cellular metabolite detection ▲ Food contaminant detection using labeled receptors
Electrochemical [63,64,65,66,67,68]	● High sensitivity and quantitative output ● Works with enzymatic, catalytic, or immuno-recognition layers ● Low sample volume and fast response ● Handheld readers easily integrated	■ Electrode fouling in biological fluids ■ Drift from surface aging or humidity ■ Requires careful calibration and reference electrodes	▲ Point-of-care diagnostics (e.g., glucose, electrolytes) ▲ Environmental heavy-metal detection ▲ Multiplexed antibiotic or metabolite sensing
Instrument-free (distance-based, time-based) [73,74,75,76]	● Requires no electronic or optical reader ● Robust in low-resource settings ● Intuitive: signal converted to flow length or time	■ Limited dynamic range ■ Sensitive to temperature, evaporation, paper porosity ■ Low resolution for fine quantification	▲ Field-ready tests for toxins, aflatoxins, or analytes ▲ Semi-quantitative screening where cost and portability dominate
Raman/SERS-based [77,78,79,80]	● Ultra-high sensitivity (down to fg–pg levels) ● Label-free molecular fingerprinting ● Discriminates bacterial strains and structural isomers	■ Expensive substrate preparation ■ Reproducibility dependent on nanoparticle hotspot density ■ Requires spectrometer	▲ Identification of pathogens ▲ Early biomarker detection (neurodegenerative proteins) ▲ Trace contaminants in water, serum, or food
Luminescent [81,82,83,84]	● No excitation source required ● High signal-to-noise ratio ● Suitable for continuous-flow reactions	■ Short emission lifetime ■ Reagent instability in humid paper matrices ■ Integration with microchannels can be complex	▲ Detection of enzymatic activity ▲ Oxidative stress or ROS analysis ▲ Signal amplification without optics
Mass spectrometry-assisted µPADs [86,87]	● Direct molecular identification with no labels ● High specificity and multi-analyte capability ● Tolerant to complex samples	■ Requires external instruments ■ Sample prep and paper substrate compatibility issues ■ Higher operating cost	▲ Forensic toxicology ▲ High-value metabolomics ▲ Confirmatory diagnostics

## 4. Applications of μPAD

Applications of μPADs evolve naturally based on the demands of each sample type. The μPADs are particularly suitable for rapid screening in resource-scarce areas due to their ease of integration, low cost, biocompatibility, and pump-free operation. In human biofluids, small drops of blood, urine, or sweat are guided through patterned cellulose, and subtle electrochemical or optical shifts reveal metabolic imbalance or infection, often within minutes. Food testing uses the same technology to look for contaminants and spoilage. Biogenic amines, or volatile compounds, manifest as distinct colors or current signatures that inspectors can immediately interpret. Environmental monitoring benefits from paper integrated with gas- or ion-responsive coatings, allowing airborne toxins or waterborne contaminants to be followed in real time. In agriculture and forensic surveillance, compact μPAD formats map pesticide residues or trace illicit chemicals, allowing inspectors to reach a firm conclusion in the field without carrying laboratory equipment.

### 4.1. Human Biofluid Diagnosis

Within patterned paper microchannels, human biofluids leave quiet analytical signatures—subtle changes in hue, conductance, or fluorescence—gradually surfacing from the cellulose lattice [88,89,90]. The same porous scaffold guides micron-scale flow without external actuation, permitting direct assays on whole blood, serum, sweat, urine, saliva, and respiratory secretions [91,92,93,94,95,96,97,98,99,100,101,102]. In these contexts, the analytical burden is shared across fluid types: high protein content, variable ionic strength, and target dilution collectively dampen signal contrast, placing a premium on interfaces that decouple transduction from matrix variability. In chronic kidney care, paper-based microfluidic platforms combine hemofiltration and color development to measure creatinine and blood urea nitrogen in plasma. The result makes it possible to quickly identify the BUN/CRE ratio at the bedside, which is in line with standard clinical benchmarks [94]. Here, hemofiltration is not a peripheral preprocessing step but a structural element of the microfluidic path, converting optically and chemically heterogeneous whole blood into a plasma stream compatible with quantitative colorimetric readout. By embedding fractionation upstream of detection, the device preserves clinically meaningful ratios while avoiding centrifugation, a design logic transferable to other blood-borne metabolites.

Similar channels host catalytic nanocomposites for drug monitoring, where cytochrome P450–based electrodes quantify propofol in serum over therapeutic ranges while suppressing interference from co-existing metabolites [93]. Electrochemical interrogation of prostate-cancer–associated miRNA-141 in synthetic urine demonstrates another direction: gold inkjet-printed electrodes and tailored DNA capture probes deliver femtomolar sensitivities and a broad linear span important for early cancer evaluation [91]. In this case, sensitivity emerges from architectural restraint rather than aggressive amplification. The gold–paper interface confines electron transfer to a chemically defined surface, limiting nonspecific adsorption from urinary constituents and stabilizing probe–target hybridization. Such electrode–matrix decoupling is essential when nucleic acids are present at trace levels in dilute yet compositionally complex fluids.

A separate line of development focuses on circulating immune markers and vesicular cargo. Carbon-dot paper substrates accumulate allergen-specific IgE directly from serum, and the successive binding events shift the fluorescence balance between emissive layers [97]. The gain in detectability arises from physical accumulation within the cellulose network, where filtration concentrates low-abundance antibodies before signal generation. This coupling of enrichment and readout reduces reliance on multi-step amplification chemistries and establishes a general strategy for immunoglobulins present near clinical decision thresholds. MXene–gold aptamer junctions recognize exosomes with composure, their hybrid metallic nodes holding a stable electrochemical tone even inside dense patient-derived fluid [95]. Here, hybridization between MXene sheets and gold nanoparticles provides both high surface area and sustained conductivity, allowing intact vesicles to be interrogated without signal drift. Stability under prolonged exposure to protein-rich serum defines a critical requirement for liquid-biopsy-oriented μPADs, where fouling often limits reproducibility. Short-wave infrared paper assays approach glucose from another angle, isolating the 6599 cm^−1^ band in serum and revealing anomeric transitions shaped by protein–glucose interactions [96]. Across these settings, μPADs navigate dense biochemical backgrounds without elaborate handling, engaging metabolism, infection, and oncologic screening [88,89].

**Figure 3:**
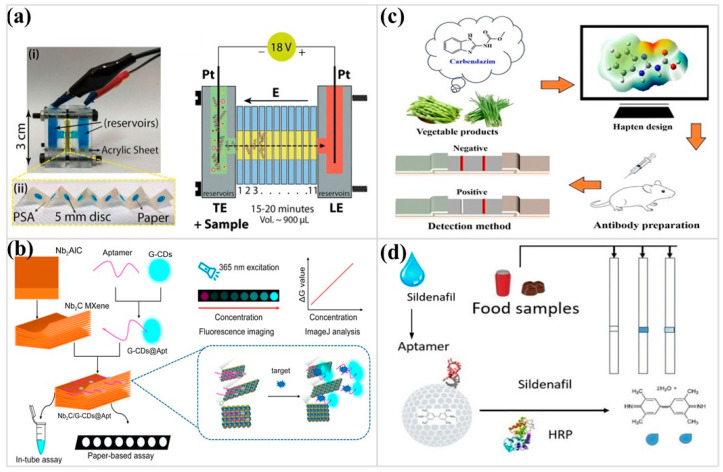
(**a**) Schematic and photo illustration of the paper-based isotachophoresis sample preparation module for nucleic acid amplification tests. (Reprinted with permission from ref. [101]. Copyright 2025 RSC). (**b**) Schematic diagram of an aptamer-based fluorescent paper sensor for detecting the SARS-CoV-2 spike protein. (Reprinted with permission from ref. [102]. Copyright 2025 MDPI). (**c**) Schematic illustration of the detection processes for carbendazim in vegetable products using a gold nanoparticle-based lateral flow immunoassay (Reprinted with permission from ref. [110]. Copyright 2025 MDPI). (**d**) Schematic illustration of the paper-based aptasensor assay for detection of the food adulterant sildenafil. (Reprinted with permission from ref. [107]. Copyright 2024 MDPI).

Wearable paper-based devices bring paper sensing directly to eccrine sweat. Intercalated electrodes fashioned from MXene sheets and metal–organic frameworks present dense catalytic surfaces, allowing micromolar lactate to be read without enzymes [98]. Three-dimensional paper scaffolds place flexible electrochemical arrays inside PDMS-sealed channels, tracking multiple sweat constituents during motion [99]. Compact microfluidic patches add another layer of practicality, combining rapid sweat uptake with pH, sodium, and uric acid readouts, while wireless links deliver signals continuously during on-body operation [100]. Beyond metabolites, paper matrices serve as biochemical concentrators. Isotachophoresis in cellulose speeds up the breakdown of bacteria and the clumping of DNA from saliva, serum, and urine. This simplifies preparation and enhances nucleic acid yields at clinically significant pathogen loads (Figure 3a) [101]. By exploiting electrophoretic mobility rather than binding equilibria, isotachophoresis selectively excludes proteins and lipids while focusing genomic DNA into narrow zones. This reframes μPADs as active separation platforms and addresses one of the most persistent barriers in decentralized molecular diagnostics: sample preparation. Virus-associated antigens can be identified using MXene-quenched fluorescent aptamers patterned onto ester membranes; spike-protein recognition elicits prompt emission recovery in saliva and other matrices, positioning µPADs as broadly adaptable tools for decentralized screening (Figure 3b) [102]. Contrast retention under autofluorescent or enzyme-rich conditions is effectively maintained through quenching–release architectures, while aptamer-mediated recognition introduces chemical resilience and modular reconfigurability. These attributes align closely with the practical demands of respiratory diagnostics, where operational robustness frequently carries greater weight than peak analytical sensitivity. A comparative overview of μPAD implementations for human biofluid analysis is provided in Table 3.

### 4.2. Food Safety Analysis

Food analysis conducted on paper rarely resembles a clinical exercise. Cellulose tolerates grease, sugar, and particulate debris without protest, a practical indifference that underlies its continued relevance. Recognition chemistries—aptamers, antibodies, enzymes, and imprinted polymers—reside within the fibrous network, remaining inactive until the target molecule arrives [103,104,105,106,107,108]. Colorimetric formats persist not because of sophistication, but because the response emerges before digital interpretation becomes necessary. In food-safety contexts, μPAD performance is governed less by nominal detection limits than by the degree to which chemical recognition can be insulated from matrix complexity. Lipids, proteins, pigments, and suspended solids actively reshape diffusion pathways, dampen contrast, and encourage non-specific adsorption rather than serving as passive backgrounds. Consequently, effective food-oriented μPADs converge on three shared priorities: decoupling of the matrix, engineered selectivity, and signal normalization under field conditions.

Nitrite detection in oil-rich foods illustrates this logic clearly. Lipid-induced mass-transfer resistance and optical interference compromise direct capillary transport. Electromembrane extraction resolves this bottleneck upstream, selectively transferring ionic nitrite into an aqueous phase under an applied electric field, after which on-paper color analysis proceeds with restored sensitivity and reproducibility [109]. The same pretreatment principle extends naturally to other polar or ionic species embedded in fatty matrices, offering a general route beyond samples amenable to direct wicking. Carbendazim assays impose an even stricter constraint. Here, hapten geometry is deliberately shaped to suppress cross-reactivity before immobilization occurs, anchoring assay performance to regulatory thresholds rather than exploratory screening (Figure 3c) [110]. In food μPADs, selectivity is not an outcome to be optimized post hoc; it is a design condition that must be established at the level of molecular recognition.

Food-focused μPADs often reveal details that conventional laboratory assays overlook. Brewers, for instance, track dimethyl sulfide by sampling the beer headspace with a color-treated paper patch; a smartphone reads the change and provides values that mirror sensory thresholds, eliminating the need for chromatographic hardware [111]. Iron is approached differently: a short immersion on reagent-prepared strips is enough to return a clear estimate, a feature that suits routine checks across varied foods [112]. In this setting, assay reliability is anchored in how reactions proceed and colors settle, rather than in the pursuit of ever-lower detection limits. Strip formulations are adjusted so chromogenic responses emerge rapidly and evenly, allowing visual readout to remain consistent despite matrix variability or repeated handling during food analysis. In metallic contamination, paper fibers loaded with dual-emissive carbon dots shift their fluorescence in two directions when exposed to copper ions, a response that remains calm even in greasy or protein-rich matrices [113]. By placing an optical reference within the paper itself, ratiometric layouts absorb variability arising from background absorption, scattering, and local concentration shifts. This built-in normalization reflects a real-world fact about food analysis: reliable signals must be able to survive changes in sample composition rather than rely on tightly controlled assay conditions.

Food adulteration assays highlight how μPAD biorecognition can be shaped for specific field problems. A paper aptasensor designed for sildenafil, for example, employs graphene-selected aptamers arranged along a lateral-flow strip, allowing the device to identify illicit additives in confectionery with minimal handling (Figure 3d) [107]. Rather than pursuing maximal sensitivity, the platform prioritizes molecular discrimination among structurally similar small compounds, reflecting enforcement-driven analysis where false positives carry practical and regulatory consequences. A different strategy is used for E. coli: dual-mode paper chips integrate magneto-optic nanoparticles, enabling electrochemical impedance and fluorescence to converge, which shortens analysis time without sacrificing sensitivity when moved to real food matrices [108]. Viscous oils pose their challenges, and a dual-channel fluorescent μPAD addresses them by quantifying aflatoxin B1 and capsaicin within the same device, demonstrating steady performance in high-fat media [114]. People often use biochemical signals to monitor seafood. For example, a nanozyme combined with oxidase enzymes shows the early rise in hypoxanthine, which is a direct sign of freshness loss during spoilage [115]. Photoluminescent paper platforms work differently. They can sense cinnamic ortho-diphenols in a wide range of foods and show small changes in composition [116]. Instead of targeting a single analyte, these devices report on a chemically coherent class, enabling compositional trend analysis where nutritional or antioxidant profiles matter more than absolute concentration. Nutritional screening follows its logic, as metal–chelate μPADs quantify vitamins C and E in both aqueous extracts and organic matrices, preserving accuracy across contrasting sample types [117]. Table 3 also summarizes the various applications of μPADs in food safety testing.

**Table 3:** Representative μPAD platform for human biofluids detection and food safety analysis.

Application Field	Detection Model	Target	Detection Range & Detection Limit	Detection Time and Cost Level	Ref.
Human biofluids detection	Electrochemical biosensor	miRNA-141 (prostate cancer) (Urine)	1 fM–100 nM LOD: 2.15 fM	15 min Low	[91]
	Electrochemical nanocatalyst sensor	Propofol (Serum)	µg/mL level LOD: 0.5 µg/mL	~min Moderate	[93]
	Colorimetric μPAD	BUN/Creatinine ratio (Whole blood)	BUN: 0.1–150 mg/dL CRE: 0.04–8.0 mg/dL	4 min Low	[94]
	Aptamer immunosensor	Colorectal cancer exosomes (Serum)	50–5 × 10^4^ particles µL^−1^ LOD: 19 particles µL^−1^	− Moderate	[95]
	Fluorescent PAD	Peanut allergen- specific IgE (Serum)	ng/mL range LOD: 15.7 ng/mL	− Low	[97]
	Wearable microfluidic patch pH, Na^+^	Uric acid	Na^+^: 0–160 mM; pH: 4–8; UA: 5–160 µM	<30 s Low	[100]
	Paper isotachophoresis	Bacterial DNA (Saliva, serum, urine)	10^2^–10^3^ CFU/mL ~12× concentration	15–20 min Low	[101]
	Fluorescent aptasensor	SARS-CoV-2 spike protein (Saliva)	ng/mL range LOD: 0.067 ng/mL	~ min Low	[102]
Food safety analysis	SPE–EME integrated μPAD	Nitrite in high-fat foods	− LOD: 1.1 mg/kg	20 min Low	[109]
	Paper immunosensor	Carbendazim fungicide	− LOD: 1.8 μg/kg	10 min Low	[110]
	Smartphone colorimetry μPAD	Dimethyl sulfide in beer	5–120 µg/L LOD: 2.7 µg/L	5 min Low	[111]
	Colorimetric test strips	Fe^2+^ in food products	− LOD: 1.26 mg/kg	30 s Low	[112]
	Dual-emissive carbon-dot μPAD	Cu^2+^ ions	ng/mL level LOD: 0.17 ng/mL	5 min Moderate	[113]
	Dual-channel fluorescent μPAD	Aflatoxin B1, capsaicin	18.75–600 µM LOD: 9.67 µM	10 min Low	[114]
	Dual-enzyme colorimetric μPAD	Hypoxanthine in shrimp	− LOD: 18.4 nM	10 min Low	[115]
	Photoluminescent paper platform	Cinnamic ortho-diphenols	µM range LOD: 3.0 µM	10 min Low	[116]
	Metal-chelate μPAD	Vitamins C and E	Vit. C: 4.4–35 mg/L; LOD: 3.1 mg/L Vit. E: 50–200 mg/L	15 min Low	[117]

### 4.3. Environmental Monitoring

**Figure 4:**
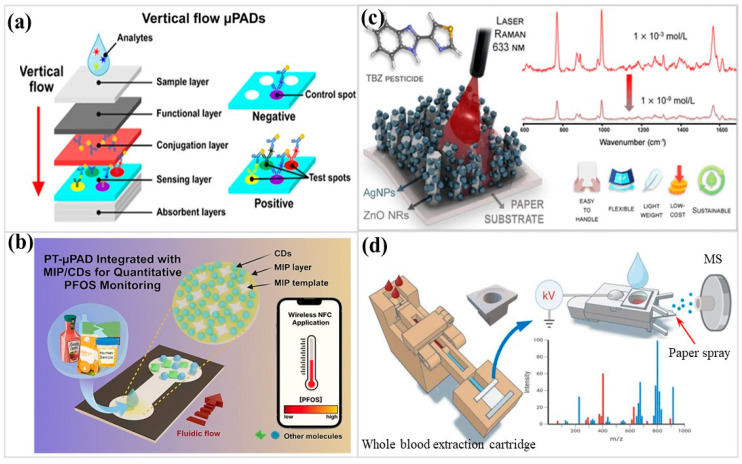
(**a**) Schematic illustration of the paper-based vertical flow assays for in vitro diagnostics and environmental monitoring. (Reprinted with permission from ref. [118]. Copyright 2025 ACS). (**b**) Schematic diagram of the photothermal μPAD integrated with carbon nanomaterials and MIP for perfluorooctanesulfonate detection. (Reprinted with permission from ref. [128]. Copyright 2025 ACS). (**c**) Schematic illustration of the ZnO nanorod/Ag nanoparticle SERS paper-based assay for the detection of environmental contaminants. (Reprinted with permission from ref. [136]. Copyright 2025 ACS). (**d**) Schematic illustration of the workflow of the whole blood extraction device using paper spray mass spectrometry. (Reprinted with permission from ref. [144]. Copyright 2025 ACS).

Environmental surveillance often unfolds in places where laboratory instruments are distant, water quality is uncertain, and decisions must be made within minutes rather than days. Microfluidic paper platforms provide an avenue for such settings because cellulose quietly manages fluid motion and supports reagents without elaborate equipment. Vertical-flow configurations demonstrate how a paper stack (Figure 4a), rather than a single plane, can handle higher sample loads and reduce detection artifacts, enabling multiplex pollutant screening in a compact format [118]. Vertical-flow designs serve more than throughput demands. By breaking lateral heterogeneity imposed by particulates, ionic fluctuations, and organic load, stacked pathways shorten transport distances and rebalance exposure, preventing saturation and hook effects so concentration trends remain legible in field measurements. In this context, three-dimensional paper routing functions as a robustness strategy rather than a purely structural refinement. Fluorescence droplet designs take one step further: when bacterial metabolism changes under toxic stress, the resulting shift in optical intensity reveals the presence of chlorophenols or heavy-metal ions with striking clarity [119]. This approach gains relevance by reading biological behavior as a whole, not by isolating individual chemicals. Metabolic changes integrate cumulative and sublethal stress that target-specific assays often miss. Spatial confinement of droplets on hydrophilic paper moderates kinetics and limits dispersion, supporting rapid, reliable early-warning assessment in environmental settings.

Certain environmental contaminants remain elusive at trace levels, slipping past routine inspection. Nanozyme-assisted colorimetric platforms translate antioxidant behavior into vivid tonal shifts that reveal oxidative burdens within chemically crowded fluids [120]. Molecular imprinting adds a more selective layer of recognition, and a triple-ratio fluorescent probe in a paper microchannel separates enoxacin from moving river water, keeping the analysis accurate even at low concentrations [121]. Here, selectivity and self-referencing operate in tandem. Molecularly imprinted cavities discriminate target molecules from structurally similar interferents, while triple-ratio fluorescence compensates for background absorption, scattering, and local concentration fluctuations. This combination transforms ratiometric signaling from a sensitivity enhancement into a mechanism for preserving analytical reliability under continuously changing flow and composition. Other platforms embrace ratiometric signaling, as demonstrated by MOF–tetracycline composites that resolve Cu^2+^ and Fe^3+^ in a single pass [122]. Smartphone imaging becomes a quiet ally, converting weak fluorescence from aptamer–metal complexes into quantified values for Pb^2+^, Hg^2+^, Cd^2+^, and As^3+^ [123]. Smartphone-assisted analysis matters less for portability than for control. Built-in normalization and pixel averaging dampen lighting fluctuations and handset variability—the dominant noise sources in field paper sensors—so reliable readout persists without laboratory optics, supporting decentralized μPAD deployment.

Some applications require precision beyond simple color changes [124,125]. Foldable paper devices married to laser-induced breakdown spectroscopy (LIBS) create their own calibration gradients, producing reliable Cu and Mn measurements in real samples [126]. Here, foldable μPADs internalize standard addition and gradient generation, stabilizing LIBS quantification against matrix variability and elevating field analysis from qualitative screening to reproducible elemental measurement. Electrochemical sensing fills gaps left by optical readouts. On-paper electrodes modified with NiFe_2_O_4_/CeO_2_, defect-rich interfaces, and coupled redox activity bind Mn^2+^ effectively, promote rapid charge transfer, and sustain quantitative stability even in ion-complex groundwater matrices [127]. Perfluorooctanesulfonate leaves a photothermal fingerprint when a molecularly imprinted layer interacts with carbon nanomaterials (Figure 4b) [128]. In the case of persistent fluorinated pollutants, photothermal transduction bypasses optical ambiguity altogether. Signal readout based on binding-driven heat release remains accessible in opaque or strongly colored samples, pointing to a growing place for non-optical transduction where visual assessment is fundamentally unreliable. Even antibiotic residues, elusive in food and aquaculture matrices, can be addressed by origami electrochemical immunosensors [129] and molecularly imprinted paper electrodes tailored to hydrochlorothiazide in wastewater streams [130]. This body of work, when taken as a whole, converges on a clear principle for environmental PADs: the analytical burden is handled at the front end—through structural design, selective chemistry, and built-in referencing—so that subsequent readout stays simple, robust, and field-legible. Table 4 summarizes representative PAD applications in environmental monitoring.

### 4.4. Pesticides and Illicit Drugs Test

Paper microdevices have become a quiet driver behind field diagnostics, especially when the target is hazardous and the sample matrix unpredictable. Pesticide detection on crops and foods often demands tools that tolerate humidity, surface roughness, and minimal preparation. Lab-on-paper formats gather considerable momentum for this purpose, integrating enzymes, aptamers, or nanomaterials into capillary-driven channels to provide immediate visual or electrochemical signals [131,132,133,134]. Exposure to omethoate causes fluorescent aptamers to dissociate from graphene oxide, reactivating emissions that are captured by smartphone imaging and interpreted using a trained neural model. Layered paper porosity, combined with convolutional neural network (CNN)-assisted analysis, attenuates fluorescence drift and stabilizes faint recovery signals, enabling quantitative readout under uneven and variable illumination in field settings [135]. Raman amplification on ZnO nanorod–silver composites gave thiabendazole a unique vibrational footprint that remained steady over repeated cycles (Figure 4c) [136]. Here, the vertical nanorod scaffold concentrates plasmonic hotspots while preserving mechanical flexibility, addressing the dual demand for spectral fidelity and conformal contact on uneven produce surfaces.

Some assays favored human intuition: carbaryl hydrolysis produced an orange band whose travel distance revealed concentration, with no calibration curve required [137]. By mapping reaction progress onto physical distance, this readout favors clarity and resilience over marginal sensitivity, a trade-off well suited to settings where instruments and user training are limited. Chloride-ion pretreatment restructures the paper–nanoparticle interface by screening electrostatic repulsion and densifying AgNP packing, thereby stabilizing plasmonic hotspots and enabling direct swab-based extraction from curved apple skins; this interfacial regulation yields uniform SERS enhancement and supports thiram detection down to 10^−8^ M under field-relevant conditions [138]. Dual-mode formulations added a visual twist: when organophosphorus pesticides disrupted MOF enzyme-mimicking activity, blue chromophores vanished and red fluorescence resurfaced, providing two cross-checking signals at once [139]. When color and fluorescence are tied together by an internal reference, misclassification driven by matrix complexity is reduced, reinforcing self-calibration as a fundamental design expectation in both illicit-substance and pesticide sensing applications.

Illicit drug tests conducted on paper occupy a distinct analytical landscape, where sampling conditions are often improvised and laboratory infrastructure is absent [140]. A forensic officer may face only saliva residues or traces left on a beverage glass. Capillary-driven paper chips have already quantified ethanol and tetrahydrocannabinol in saliva without pretreatment, a rare achievement for highly viscous biofluids [141]. Here, channel-assisted capillary acceleration mitigates mucin-induced flow resistance, stabilizing color development and preserving quantification accuracy across variable salivary viscosities. At the more hazardous frontier, paper substrates became a functional bridge between SERS and paper-spray mass spectrometry, enabling the separation of fentanyl analogs with nearly indistinguishable vibrational fingerprints inside heroin mixtures [142]. By decoupling spectral similarity from molecular mass discrimination, the dual-mode paper interface resolves false positives that single-technique screening cannot suppress.

Beyond forensic screening, drug discovery workflows have also begun to leverage paper platforms. When magnetic nanoparticles are incorporated into microdevice workflows, β-lactamase inhibitors can be pulled directly from chemically crowded botanical extracts with a speed and selectivity that conventional column chromatography rarely delivers [143]. When affinity interactions are confined within paper substrates, target capture becomes spatially focused, accelerating discovery while retaining biochemical specificity in chemically crowded matrices. For blood-borne drugs, whole-blood cartridges integrate solid-phase extraction to preconcentrate opioids, synthetic cannabinoids, and stimulants prior to paper-spray ionization, pushing detection into the low-nanogram regime (Figure 4d) [144]. Extracting embeddings before ionization changes the paper from just a surface to an active tool for cleaning up and boosting signals. In public and social settings, screening tools are judged by how quickly they respond rather than by forensic completeness. Paper platforms equipped with multiplex electrodes register ketamine, methamphetamine, and alprazolam within seconds [145], and a fingernail-worn strip reveals γ-hydroxybutyrate through selective attenuation of iron–phenanthroline color development [146]. Designed for preventive intervention, the wearable format trades instrumental precision for situational awareness at the point of exposure. Table 4 summarizes representative μPAD implementations for pesticide and illicit-drug detection, highlighting how matrix tolerance, selectivity control, and deployment context collectively shape device architecture rather than application category alone.

**Table 4:** Representative μPAD platform for environmental monitoring, pesticide, and illicit drug detection.

Application Field	Detection Model	Target	Detection Range & Detection Limit	Detection Time and Cost Level	Ref.
Environmental monitoring	Ratiometric MOF@TC fluorescence μPAD	Cu^2+^ and Fe^3+^ (drinking water)	Cu^2+^: 0.1–80 μM; Fe^3+^: 0.2–160 μM LOD: 0.027 μM (Cu^2+^); 0.019 μM (Fe^3+^)	5–8 min Low	[122]
	Colorimetric μPAD + solid-phase extraction	Phosphate (water and soil)	0.05–1 mg/L LOD: 0.089 mg/L	10 min Low	[124]
	Foldable LIBS-coupled μPAD	Cu and Mn (water)	mg/L level LOD: 924 μg/L (Cu); 890 μg/L (Mn)	15 min Medium	[126]
	Electrochemical μPAD (NiFe_2_O_4_/CeO_2_)	Mn^2+^ (groundwater)	2–8 mg/L LOD: 1.72 mg/L	5 min Low	[127]
	Photothermal μPAD with MIP–carbon nanomaterials	PFOS (PFAS) (water, food, and biological samples)	1.5–7.0 pg/mL LOD: 7.0 fg/mL	5 min Medium	[128]
	Origami electrochemical immunosensor	Quinolone antibiotic residues (milk, honey, fish)	0.01–10 μg/mL LOD: 2.02 ng/mL	10 min Low	[129]
	Electrochemical μPAD (MIP–rGO)	Hydrochlorothiazide pollution (wastewater and aquatic)	5–100 μmol/L^−^ LOD: 1.8 μmol/L	5 min Low	[130]
Illicit drugs test	Smartphone-integrated MOF colorimetry	Glyphosate (pesticide)	0.02–40 μg/mL LOD: 1 ng/mL	15 min Low	[133]
	Aptamer–GO fluorescence	Omethoate (pesticide)	0–750 nM −	15 min Low	[135]
	ZnO/Ag SERS substrate	Thiabendazole (pesticide)	10^−8^–10^−4^ M LOD: 5.0 × 10^−10^ M	~ min Low	[136]
	Distance-based colorimetric	Carbaryl (pesticide)	70–110 ng/mL LOD: 20 ng/mL	10 min Low	[137]
	Dual-mode MOF colorimetric/fluorescent	Organophosphorus pesticides	− LOD: 1.04 ng/mL	15 min Low	[139]
	SERS + paper-spray MS	Fentanyl and analogs (drugs)	mg/mL level LOD: 34 μg/mL (SERS); 0.32 μg/mL (PS-MS)	5 min Medium	[142]
	Colorimetric + MNP affinity	Drug-screening inhibitors (drug)	2.5–20 μg/mL −	5 min Low	[143]
	Paper-spray MS with SPE	Illicit drugs in blood	0.1–1000 ng/mL LOD: 4 ng/mL	3 min Medium	[144]
	Wearable fingernail μPAD	γ-hydroxybutyrate (drug)	LOD: 0.55 μg/mL (digital); naked-eye 10 mg/mL	15 min Low	[146]

## 5. AI Applications

### 5.1. AI-Assisted Design and Fabrication Optimization

**Figure 5:**
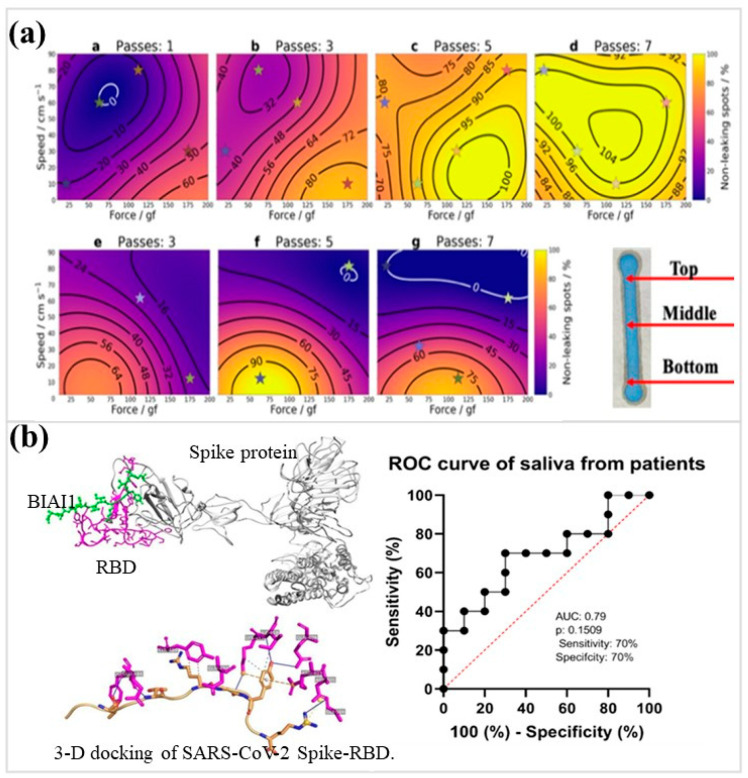
(**a**) Using machine learning, obtained contour maps of drawing speed, applied force, and number of passes for a paper-based device. (Reprinted with permission from ref. [147]. Copyright 2024 ACS). (**b**) Schematic illustration of the bio-inspired peptide for salivary detection of SARS-CoV-2 with machine learning algorithms. (Reprinted with permission from ref. [153]. Copyright 2025 MDPI).

AI has been increasingly embedded upstream of analysis, where it optimizes fabrication variables that are otherwise difficult to balance experimentally. Machine-learning–guided control of printed hydrophobic barriers enables simultaneous tuning of solvent resistance, channel integrity, and mechanical durability, reducing trial-and-error iterations during μPAD fabrication (Figure 5a) [147]. Machine-learning–guided parameter tuning increased non-leakage yields to >90%, narrowed channel-width deviation below 10%, and markedly reduced fabrication variance relative to manual trial-and-error control. Beyond two-dimensional layouts, simulation-driven AI frameworks integrate fluid dynamics, nanoparticle amplification behavior, Bayesian classification, and receiver operating characteristic (ROC) analysis to pre-validate 3D origami paper diagnostics, ensuring target performance before physical prototyping [148]. Simulation-validated origami μPADs achieved tenfold sensitivity enhancement, lowering detection limits from ~0.01 to ~0.001 μg/mL while maintaining area under the curve (AUC) values above 0.97 under noise. In these situations, AI indirectly improves detection by making sure that flow, reaction timing, and signal uniformity are all stable across devices.

### 5.2. AI-Enhanced Signal Processing and Noise Suppression

AI intervention typically operates at the level of signal interpretation rather than sensor chemistry, reshaping weak or drifting responses into consistent analytical outputs. In agri-food μPADs and paper-based gas sensors, colorimetric and electrochemical signals are reassessed algorithmically, which improves tolerance to matrix variability and multi-analyte interference [149]. The same approach stabilizes faint chemiresistive expansions, enabling reliable real-time monitoring under conditions where raw signals would otherwise fluctuate or decay [150]. YOLOv5-assisted vision models turn colorimetric sweat tests into quantitative biomarker values at the user interface level. They keep high recognition accuracy even when the lighting and camera hardware change [151]. Specifically, object-detection–guided color extraction achieved a mean average precision of 99.5%, while cross-device and illumination variability no longer propagated into concentration readouts, reducing image-derived quantification error relative to manual RGB thresholding. AI CNNs extract spatiotemporal features from pressure signals for wearable paper sensors. This approach markedly reduces misclassification in breathing and pulse recognition relative to threshold-based methods [152]. Substituting fixed decision thresholds with CNN-driven feature extraction raised recognition accuracy to 98.33%, reducing noise-related misclassification and preserving stable decoding of respiratory and pulse signals despite ongoing mechanical disturbance.

### 5.3. AI-Driven Data Interpretation and Pattern Recognition

When μPAD outputs exceed what visual inspection can resolve, algorithmic interpretation becomes critical. In saliva-based infection detection, support vector machines (SVM) and neural network classifiers are used to decode computer-simulated peptide receptors, thereby enabling the differentiation of weak and overlapping electrochemical features (Figure 5b) [153]. This method achieves approximately 90% diagnostic accuracy and 100% sensitivity, effectively avoiding false negatives that occur during peak manual reading periods. Food spoilage assessment has moved away from subjective visual checks toward data-driven classification. Using random-forest models, RGB signatures produced by bacterial metabolites are interpreted with over 95% accuracy, reducing operator bias and maintaining consistent decisions despite changes in illumination or dye response drift [154].

Fluorescent μPAD arrays based on multicolor quantum dots further exploit machine learning to distinguish bacterial species across orders of magnitude in concentration while simultaneously reporting antimicrobial effects [155]. Here, machine-learning pattern recognition enabled species identification across 10^3^–10^7^ CFU mL^−1^, maintaining high accuracy where single-channel fluorescence thresholds collapse under signal overlap. Dimensionality reduction and discriminant analysis also allow paper-based electronic sensors to classify solvent compositions with full accuracy, replacing labor-intensive reference methods such as Karl Fischer titration [156]. With linear discriminant analysis, complete class separation was obtained together with sub-ppm resolution in water measurement, removing errors introduced by titration steps and suppressing variability between individual analysts. Ratiometric phosphorescent patterns, when read by smartphones, translate minor spectral imbalance into chemically specific fingerprints. At micromolar levels, closely related antibiotics and their mixtures can be distinguished without the cross-interference that constrains conventional single-channel assays [157].

## 6. Critical Challenges and Future Perspectives

Microfluidic paper devices invite attention because cellulose seems effortless: light, absorbent, and self-driven by capillary pull. The reality is harsher. Fibers shift with humidity, tighten or loosen without warning, and defy neat channel layouts. Patterning—whether through wax infusion, laser etching, or printed varnish—attempts to tame this behavior rather than replace it. Even so, fibers expand or compress with humidity, wax redistributes unevenly, and laser etching may carbonize surfaces that later weaken enzyme activity. The lesson emerging from fabrication studies is not about adding more technology to paper but about designing around its material temperament: forming hydrophobic borders that tolerate solvent creep, embedding conductive networks that flex with the substrate, and accepting that channel uniformity arises from process tuning rather than ideal drawings [24,26,35,37,42,49,50]. A central limitation lies in the irreversibility of paper heterogeneity. Fiber swelling, pore collapse, and wax redistribution do not merely introduce noise; they permanently alter capillary pathways after initial wetting. As a result, identical μPAD geometries may diverge in flow timing and reagent exposure across uses, complicating calibration transfer and long-term storage. Future robustness depends on constraining these irreversible microstructural shifts rather than refining nominal channel designs [1,2,158]. These recurring material and signal instabilities unify earlier discussions on fabrication, detection, and applications, clarifying why robustness—rather than novelty—now defines progress in μPAD development.

Detection methods reveal a different sort of limitation. A single paper zone may host complex interactions, such as enzyme kinetics, molecular imprinting, and photoexcitation; however, the signal must remain discernible under field conditions. Colorimetric μPADs remain accessible, but heavy matrices such as blood, fatty foods, or plant extracts darken backgrounds and compress dynamic range [4,16,25,56]. Fluorescence and ratiometric probes reduce ambiguity, although porosity and humidity soften emission intensity and shorten reagent lifetimes [7,8,26,28,69]. Electrochemical sensing resists optical interference, but electrode fatigue, ion migration, and swelling can distort calibration curves across storage cycles [5,6,9,21,63]. Signal stability will depend on hybrid designs that include optical and electrochemical readouts housed in separate layers, nanocatalysts anchored inside cellulose pores, or distance-based valves that convert analyte content into controlled wicking fronts [73,76]. Signal instability in μPADs is rarely chemical in origin alone. Instead, it emerges from coupled drift between substrate hydration, reagent redistribution, and transduction interfaces. Optical fading, electrode baseline shifts, and distance-encoded variability often share a common cause: uncontrolled fluid–material interaction over time [159]. Addressing such an issue requires separating sensing chemistry from transport instability, rather than compensating downstream through calibration or normalization.

Applications expose the most unforgiving constraints. Environmental monitoring asks devices to interpret fluctuating ionic loads, pH swings, and microbial signatures while submerged or exposed to dust [10,11,25,118]. Clinical samples bring complications that seldom appear in controlled assays: ruptured blood cells shed chromophores, salivary enzymes quietly dismantle recognition layers, and exosomal cargo loosens antibody films during routine transport [12,13,23,110]. Food substrates add another layer of difficulty, mixing fats, dyes, and preservatives that mask optical contrast [15,18,28,105]. Agricultural detection of organophosphates or neonicotinoids may be performed on leaves, skin, or market produce, where humidity and surface roughness erode assay reproducibility [20,21,25,131]. Illicit drug surveillance adds a psychological burden: the device must deliver confident answers from a sip of saliva or a beverage glass, without centrifugation or protective labware, and often within minutes [12,21,140,143]. Across applications, failure rarely stems from target chemistry, but from how uncontrolled matrices amplify small material and transport instabilities into decisive analytical errors. Field reliability will rely on microfluidic buffering—sacrificial pre-wick zones, printed membranes that trap pigments, or origami compartments that separate capture and readout regions before the user opens the device.

AI shifts the discussion from fabrication and chemistry to interpretation. Raw pixels, gradients, and shadow artifacts once considered “noise” are now analytical substrates. Computer vision routines decode lateral-flow zones, distance bars, and dual-color fluorescence, reconstructing concentration profiles even when reagent coverage is incomplete [8,25,149,150]. Machine learning has begun to influence design itself; hydrophobic barrier width, solvent tolerance, and electrode geometry can be optimized algorithmically, shortening prototyping cycles and revealing fabrication parameters invisible to intuition [147]. In complex biofluids, AI classifiers discern spectral changes linked to peptides, antibiotics, or glucose anomers, reducing reliance on lab-based reference assays [14,25,155,157]. Neural architectures trained on tactile signatures interpret pressure pads, breathing traces, or sweat flux on wearable μPADs, quietly translating physiological rhythms into metrics that no human observer could extract reliably [152]. AI refines data interpretation, but intrinsic physical uncertainty remains. Algorithms trained to offset illumination changes, fabrication variability, or matrix interference presuppose that such disturbances stay within a stable statistical domain. When material behavior drifts beyond this boundary—owing to aging, humidity fluctuations, or previously unencountered samples—predictive robustness deteriorates rapidly [21,160]. Progress in μPADs therefore depends on synchronizing algorithmic compensation with material stabilization, rather than positioning AI as an all-encompassing corrective solution. The next generation of devices may embed model inference on edge sensors, moving beyond smartphone readouts and toward μPADs that reason about context, uncertainty, and historical variability in real time [148].

Collectively, these challenges indicate that μPAD progress is constrained less by missing functionalities than by unresolved couplings between material behavior, signal generation, and interpretive models under realistic conditions. μPADs transform cellulose into an analytical landscape where capillary flow, patterned barriers, and embedded electrodes collaborate to stabilize reactions at microliter scales. Detection expands from color shifts to hybrid electrochemical–optical readouts that stay coherent in blood, oils, and polluted water. Signals drawn from paper devices become most persuasive when confronted with unruly samples: electrolytes shifting in serum, pesticide traces on leaf surfaces, metals dissolved in runoff, or adulterants lurking in foods. Cellulose fibers decide how those encounters unfold, sometimes slowing absorption, sometimes sharpening reaction fronts. Channel design has drifted from waxed outlines to laser trenches and printed reliefs that endure humidity. Optical and electrochemical readouts seldom compete; they lean on each other during fieldwork. AI gradually enters this ecosystem, refining gradients, interpreting faint color fields, and guiding device geometry, allowing disposable paper platforms to approach the confidence of laboratory instruments.

## Figures and Tables

**Table 1:** Comparative overview of μPAD fabrication methods.

Fabrication Technique and Ref.	Advantages	Limitations	Representative Applications	Suitable Paper Materials and Ref.
Wax printing [1,2,34]	Very low cost; rapid prototyping; minimal equipment	Barrier spreading; limited spatial resolution; heat-induced deformation	Educational labs; single-assay diagnostics	Filter paper; Chromatography paper
Inkjet/chemical Deposition [3,4,25]	High precision; solvent compatibility; sharp channel edges	Requires post-treatment; reagent loading variability	Multiplexed assays; MOF-functionalized biosensors	Glossy paper; Nitrocellulose membrane; Chromatography paper
Laser cutting/ knife crafting [1,21,36]	Seconds-scale fabrication; Controllable microgrooves; mass-scalable	Localized carbonization; high power may damage substrates	Time-regulated microfluidics; delay/acceleration control	Filter paper; Paper towel
Origami/ Lamination [1,28,36]	No adhesives; compact multilayer structures	Alignment complexity; limited structural rigidity	Multistep assays; vertical flow μPADs	Office printing paper; Chromatography paper
Printed/embroidered Electrodes [12,25,36]	Flexible and wearable; replaceable sensing units	Mechanical fatigue; electrode drift	Wound pH sensing; wearable analytics	Filter paper; Cellulose paper; Paper towel
Actuator-integrated μPADs [1,31,36]	Conformal contact; robotic sampling	Design complexity; energy requirements	Curved-surface sensing; biomedical actuation	Cellulose paper; Chromatography paper
Vapor-phase Hydrophobization [2,18,46]	Equipment-free, simple	Barrier variability; humidity effect	Multiplexed assays; clinical biological testing	Cellulose paper; Nitrocellulose; Chromatography paper
Electrochemical nanomaterial integration [4,35,44]	High sensitivity, selectivity	Drift, long-term stability	Multiplexed assays; functional nanomaterial sensors	Cellulose paper; Filter paper; Nitrocellulose

## Data Availability

The data presented in this study are available upon request from the corresponding author.
